# Cure of Stammering

**Published:** 1846-04

**Authors:** 

**Affiliations:** D’Uzès


*Cure of Stammering.* By Dr. Serre (d'Uzès.)—Dr. Serre's communication excited an unusual degree of interest, due to the circumstance, that the learned essayist had struggled since his youth against the infirmity to which he this day directed the attention of the Academy ; and, certainly, the distinct enunciation and unhesitating utterance of the speaker were the most striking proofs of the correctness of his views on the subject. The unfortunate results of the surgical operations proposed, not long since, for the cure of stammering, no less than the disgraceful charlatanry to which they were made subservient, threw them into well merited discredit. The method now brought forward does not bear the same character ; it is purely the result of philosophical induction, and rests upon the perseverance and reason of the patient. The following precepts are laid down by Dr. Serre :—1. Stammering, like other defects of speech, can only be got rid of by a firm and persevering determination. 2. Equisyllabism, or the enunciation at equal intervals of each syllable of every word, is the first rule to which the stammerer must submit ; under its influence regularity takes forcibly the place of that disorder which characterises his utterance. 3. The action of the orator not only constitutes a language completive of vocal expression, but serves, also, according to Dr. Serre, to modulate the sound emitted, the volume and energy of which is instinctively regulated by the precision of the accompanying gesture. 4. The frequent exercise of equisyllabic pronunciation combined with appropriate action must infallibly restore the function of speech to its natural condition. Stammering is certainly so often the result of bashfulness, that the rules laid down by Dr. Serre cannot fail in producing a favourable change in most persons afflicted with the infirmity. It is obvious that they must, on the contrary, prove inefficient when some physical obstacle exists to a proper enunciation. Such instances are, however, comparatively rare, and to all others Dr. Serre's method is immediately applicable.—*Ibid.*

